# A Pheromone-Inspired Monitoring Strategy Using a Swarm of Underwater Robots

**DOI:** 10.3390/s19194089

**Published:** 2019-09-21

**Authors:** Guannan Li, Chao Chen, Chao Geng, Meng Li, Hongli Xu, Yang Lin

**Affiliations:** 1 State Key Laboratory of Robotics, Shenyang Institute of Automation, Chinese Academy of Sciences, Shenyang 110016, China; 2 Institutes for Robotics and Intelligent Manufacturing, Chinese Academy of Sciences, Shenyang 110016, China; 3 University of Chinese Academy of Sciences, Beijing 100049, China; 4 Shenzhen Institutes of Advanced Technology, Chinese Academy of Sciences (CAS), Shenzhen 518055, China; 5 School of Information Science and Engineering, Northeastern University, Shenyang 110819, China; 6 The School of Information and Science Technology, Zhejiang Sci-Tech University (ZSTU), Hangzhou 310018, China

**Keywords:** swarm robotics, underwater robot, virtual pheromone, marine monitoring, target search

## Abstract

The advent of the swarm makes it feasible to dynamically monitor a wide area for maritime applications. The crucial problems of underwater swarm monitoring are communication and behavior coordination. To tackle these problems, we propose a wide area monitoring strategy that searches for static targets of interest simultaneously. Traditionally, an underwater robot adopts either acoustic communication or optical communication. However, the former is low in bandwidth and the latter is short in communication range. Our strategy coordinates underwater robots through indirect communication, which is inspired by social insects that exchange information by pheromone. The indirect communication is established with the help of a set of underwater communication nodes. We adopt a virtual pheromone-based controller and provide a set of rules to integrate the area of interest into the pheromone. Based on the information in the virtual pheromone, behavior laws are developed to guide the swarm to monitor and search with nearby information. In addition, a robot can improve its performance when using additional far-away pheromone information. The monitoring strategy is further improved by adopting a swarm evolution scheme which automatically adjusts the visiting period. Experimental results show that our strategy is superior to the random strategy in most cases.

## 1. Introduction

The development of the science and technology of robots makes it feasible to produce large quantities of robots with low cost. Organizing these robots to work together has become a hot topic in the research community and recent years have witnessed rapid progress in robot swarms. Nature is one of the best sources for swarm intelligence, and many communication mechanisms have been developed based on nature and animal behavior [1,2,3]. Social insects usually adopt two communication schemes, i.e., direct communication and indirect communication. Insects can exchange information via direct communication. For example, bees can indicate the positions of nectar source through waggle dance [4]. On the other hand, some insects adopt indirect communication by secreting pheromone into the environment and other insects can get the message by sensing the pheromone.

By communicating with each other, swarm robots can work more efficiently than a single robot. In addition, a swarm shows advantages in terms of robustness, scalability, and flexibility. The number of an Unmanned Aerial Vehicle (UAV) swarm has reached hundreds or even thousands [5,6], and the UAV swarm has been widely used in light shows [5,6], military applications [7] and area surveillance [8]. Unmanned Ground Vehicle (UGV) groups have been applied to transport objects in ports [9], sort parcels [10] and perform in various shows. For example, a spectacular show in the Pyeongchang Winter Olympics was performed by a UGV swarm [11].

Compared with UAV and UGV swarms, the development of an underwater robot swarm encounters a bottleneck, i.e., the lack of proper communication methods, especially for swarms consisting of a large number of underwater robots that travel in an extensive area. For UAVs and UGVs, they can communicate via radio, which can transmit large volumes of data in a short time. Because of attenuation, it is challenging to adopt radio communication in an underwater environment. Available underwater peer-to-peer wireless communications are mainly carried out through acoustic and optical waves. However, the acoustic communication is low in bandwidth and is bothered by multipath propagation, frequency and temporal fading, while the optical communication is short in range. The lack of a proper direct communication method makes it difficult to organize underwater swarms with a large number of robots. As a result, current underwater multi-robot systems usually contain a limited number of robots [12]. In [13], the swarm consists of a larger amount of robots, but it fails to cover an extensive region due to the limitation of adopted optical communication.

In order to efficiently accomplish assigned tasks, various types of research have been performed to control swarms’ behaviors, of which some focus on monitoring behavior. For example, UAV and UGV swarms have been extensively used for an area monitoring [14]. In some cases, the swarm reports hazards when they are detected in the monitoring process [15]. When it comes to marine environmental and hazards monitoring, an Unmanned Surface Vehicle (USV) swarm has been widely adopted [16,17,18,19,20]. Underwater robot swarms have been attracting much interest recently, such as the COCORO [13,21] and MONSUN-II projects for swarm missions [22].

In this paper, the problem we aim to solve is how to coordinate a swarm of underwater robots via indirect communication. We target an underwater robot swarm consisting of 10–100 robots, which monitors an extensive area of 10–100 km${}^{2}$. Direct communication is inappropriate in this case: radio and optical light based methods are short in range, unable to cover the large area; acoustic communication is too costly to be applied to a swarm because it costs around 50,000 dollars to adopt an acoustic modem on one robot. Moreover, when many robots communicate simultaneously, it is challenging to deal with communication conflicts. Together with other factors, such as multipath propagation, frequency and temporal fading and moving effects, it is infeasible to coordinate such a large swarm using direct communication. As a result, we look into indirect communication in this paper.

We propose a wide area monitoring strategy for an underwater swarm that takes account of the communication and behavior coordination. The main contributions are as follows:

- We propose a communication network to organize a swarm of underwater robots using indirect communication. The network consists of a set of underwater communication nodes. There are various underwater navigation methods—such as Terrain-Referenced Navigation (TRN) [23], Database-Referenced Navigation (DBRN) [24] and Gravity Aided Navigation (GAN) [25]—for an underwater robot to periodically visit the nodes to exchange information and charge batteries if needed.
- We apply a pheromone-based controller to coordinate a swarm to monitor marine environment and search for static targets on the seafloor. The controller is composed of two layers: the layer of virtual pheromone and the layer of behavior laws. Virtual pheromone indicates the pheromone density in the area of interest (AOI). An algorithm is developed to map an AOI of a random shape to the virtual pheromone in the form of a matrix. Behavior laws are designed on top of the virtual pheromone, such that a swarm continuously monitors the environment. During the monitoring process, the swarm can also search for and report specific static targets, such as hazards or wreckage. Note that the controller is bio-inspired, and thus we do not prove the convergence of adopted algorithms.
- We introduce a swarm evolution scheme to improve the monitoring strategy by automatically adjusting the robots’ visiting period. Experimental results indicate that the choice of a visiting period affects a swarm’s performance. After adopting an evolution scheme, a swarm can achieve an acceptable performance by avoiding unfavorable cases.

The rest of this paper is organized as follows. Related work is discussed in Section 2. We describe the problem and introduce the pheromone-based controller in Section 3. The pheromone map is explained in Section 4. The behavior laws to monitor the environment and search for static targets are demonstrated in Section 5. In Section 6, we present the simulation and real-world experimental results. Section 7 concludes this paper.

## 2. Related Work

### 2.1. Underwater Communication

The problem we plan to solve is to organize underwater robots into a swarm that can work in a cooperative fashion. The cooperation foundation is to exchange information among robots so that a robot can make a decision based on the current status of other robots. For UAVs and UGVs, the communication is not considered as a serious problem because these robots can share information via radio communication. In some research, swarms of UAVs or UGVs are connected to a network so that one robot can exchange data with any other robot in the swarm [26,27]. At present, there are several methods for underwater communication. Acoustic communication is the most widely used method for communication among underwater devices. The communication range for acoustic communication can reach kilometers, but the drawback is also significant as the communication is low in bandwidth and is vulnerable to multipath propagation, frequency and temporal fading [28,29,30]. These features make the acoustic communication hard to be used to organize a robot swarm on a large scale. Optical communication is also widely used in the underwater environment, mainly with blue-green lights [31,32,33,34]. The speed of optical communication can reach the level of Mbps to Gbps, making it possible to transmit a large volume of data in a short time. However, optical communication only works at short distances. With scattered light, the communication range is limited to meters. With laser, the communication range can reach tens of meters, but it requires stable alignment [35,36,37]. Other underwater communication methods include the magnetic-based method [38,39] and the current field-based method [40,41]. However, these methods are far from applicable. Another way to exchange data among underwater robots is via underwater communication nodes. An underwater communication node is a device that is deployed on the seafloor. When an underwater robot comes to visit it and the distance between the node and the underwater robot becomes smaller than a threshold, the robot can exchange large volumes of data with the node quickly. This is because the node and the robot can communicate through optical light, physical contraction, or even radio signals [42,43]. Communication nodes can be used to build a communication network to cover an extensive area.

### 2.2. Pheromone-Inspired Robot Swarms

Pheromones have become the most popular inspiration source for robot swarms and thus we give a brief review on pheromone-inspired applications. Various social insects use pheromone-based communication to control swarm behaviors, such as forging and collective decision [44]. With pheromones, one-to-one communication is not necessary and agents in the swarm are anonymous, providing the swarm flexibility and robustness. References [45,46] introduce a cellular automata ant memory model for swarm foraging tasks. Reference [47] present cooperative and non-cooperative models for foraging by using stigmergy. Then, robots can transport the found food collectively. Reference [48] propose a foraging approach that does not depend on free parameters. In [49,50], researchers use a virtual pheromone-based method to cover a region. Levy flight is added and the effect of noise is studied. Reference [51] propose a foraging scheme with multiple nests, and a genetic algorithm is adopted to improve the performance of the method. These studies have shown the effectiveness of pheromone but failed to explain how to secrete pheromones by real robots.

Several approaches have been proposed to mimic pheromone with robots and one method is to use radio communication strategies. Reference [52] propose a swarm searching method, but it relies on real-time communication. Reference [53] organize a UAV swarm by mimicking pheromone with an ad hoc wireless network. A pheromone-inspired collective transportation method is proposed in [54], with all robots connected to a communication network. In [55,56,57], a virtual pheromone is realized with communication between a pair of robots. Reference [58] use a virtual pheromone to coordinate a swarm consisting of UAVs and UGVs, but radio is still necessary. Reference [59] show foraging with multiple nests, but radio communication is necessary. For [60], a global server is necessary to build the pheromone map. Reference [61] does not need a previously built communication network, but wireless communication is still required. However, these methods are not suitable for underwater robot swarms because of radio signal attenuation. Adopting acoustic modems for these schemes will significantly increase the cost of the swarm and encounter technology challenges.

In some research, chemical substances are used as pheromone markers [62], and alcohol is widely used to act as a virtual pheromone [63,64,65,66]. It is obvious that this idea is not suitable for an extensive underwater area. Other ideas to secrete virtual pheromone include the use of optical light [67], infrared communication [68], and LCD and XBee [69]. Nonetheless, none of them work for underwater robot swarms.

Researchers begin with mimicking the behavior of social insects and they can build wireless communication networks through radio readily. However, it is infeasible to build point-to-point direct communication for large swarms in underwater environments. This forces us to explore a swarm scheme with indirect communication. In nature, one-to-one communication is not necessary for pheromones, so we propose to mimic pheromones by allowing robots to periodically visit an underwater communication network.

### 2.3. Comparison with Available Schemes

Monitoring the environment and searching for targets is a main application for multi-robot systems. To realize this, robots involved in the swarm need to work cooperatively. There are two ways to organize the robots: a consensus way and a distributed way.

When organized into a consensus structure, the whole swarm obeys commands from a leader. To achieve this, the leader needs to be able to communicate with all robots in the swarm, so that it can gather data from robots, make decisions based on the information, and send commands to others. As the leader has access to global information of the swarm, it can make optimal decisions, such as path planning and task assignment [70]. The drawback of this strategy is that the leader is a single point of failure (SPOF) of the swarm. The failure of the leader will result in collapse of the whole swarm. To solve this problem, some methods have been proposed to enhance the robustness of the swarm using a dynamic leader, such as electing a leader with swarm decision-making methods, or dynamically changing the roles of the robots in the swarm [4].

In a distributed robot swarm, a leader is not required and robots make decisions based on the obtained information. At present, most methods are based on direct communication, e.g., [71] proposes a power-efficient system where a node communicates with others within a certain communication range. In addition, methods using indirect communication have been extensively studied, which are mainly inspired by foraging of the ant colony. To build a robot swarm with indirect communication, we assume a communication network consisting of communication nodes have been deployed into the AOI in advance. Field experiments have verified that robots are able to exchange large volumes of data in a short time by visiting a communication node. It is worth mentioning that, as the positions of communication nodes are fixed, an underwater robot can be navigated to a node by affordable methods, such as TRN [23], DBRN [24] and GAN [25].

Direction communication adopts either acoustic or optical communication, but the former is low in bandwidth and the latter is short in range, which limits the communication capability of the leader in a consensus structure so that it cannot gather needed information in time. Therefore, in this paper, we coordinate the swarm in a distributed way via indirect communication.

## 3. Problem Statement and Solution

### 3.1. Problem Statement and Underwater Robot Swarm with Indirect Communication

This paper seeks to develop a method to coordinate a large number of underwater robots into a swarm that can work cooperatively and can be applied to monitor the environment while searching for underwater targets simultaneously. The swarm is deployed into an extensive area in kilometers, which means that the distance between a pair of robots will be too far to communicate with optical lights, and acoustic communication cannot meet the communication bandwidth requirement. Thus, we abandon direct peer-to-peer communication among robots. Then, the problem can be reformulated as how to use N robots to monitor the environment and search for underwater targets in a cooperative way without direct communication.

**Figure 1:**
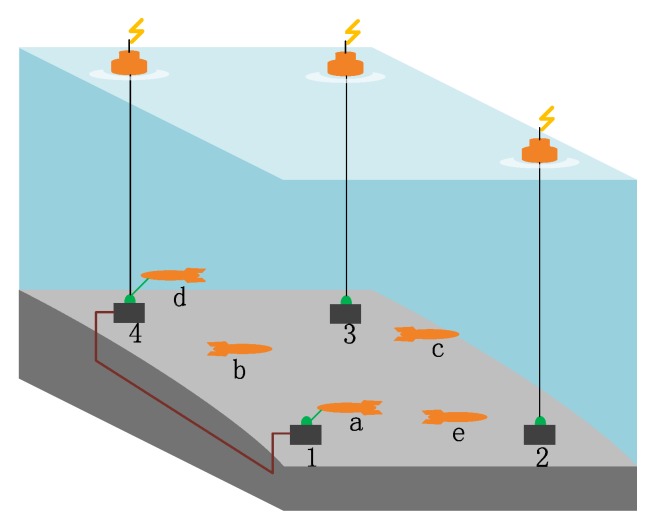
The system consists of underwater robots and communication nodes. In addition, **1**–**4** are communication nodes that have already been deployed into the AOI. Nodes 1 and 4 are connected with an underwater cable while the rest nodes are connected to buoys equipped with antennas so that they can communicate via radio signals. **a**–**e** are underwater robots equipped with an optical communication device, enabling them to exchange data with the communication network when visiting a communication node. In the figure, robot **a** is communicating with communication node 1, and robot **d** is communicating with communication node 4. Other robots, i.e., robots **b**, **c** and **e** are searching. The relationship between robots and nodes is not one-to-one, and one robot can visit any node.

Our solution is to organize an underwater robot swarm with the help of a communication network. We deploy a set of underwater communication nodes into the AOI. These communication nodes are connected through underwater cables or radio (if the communication node is connected to a buoy equipped with antennas). Finally, these nodes form an underwater communication network, and they share data as a whole. When visiting a communication node, an underwater robot can exchange data with the node through optical communication, transferring a large volume of data in a short time. The whole AOI is shown in Figure 1.

It is assumed that all robots are equipped with localization devices, and the localization error can be eliminated when visiting a communication node. Thus, we can assume that a robot can get a relatively accurate position. All robots have the information of the AOI, including the shape of the AOI and the positions of the communication nodes. Robots are also equipped with collision avoidance sensors that are able to detect the existence of another robot when the distance between them is smaller than a threshold. This device can be a sonar-based, or an optical light-based sensor [67].

### 3.2. Virtual Pheromone-Based Controller

**Figure 2:**
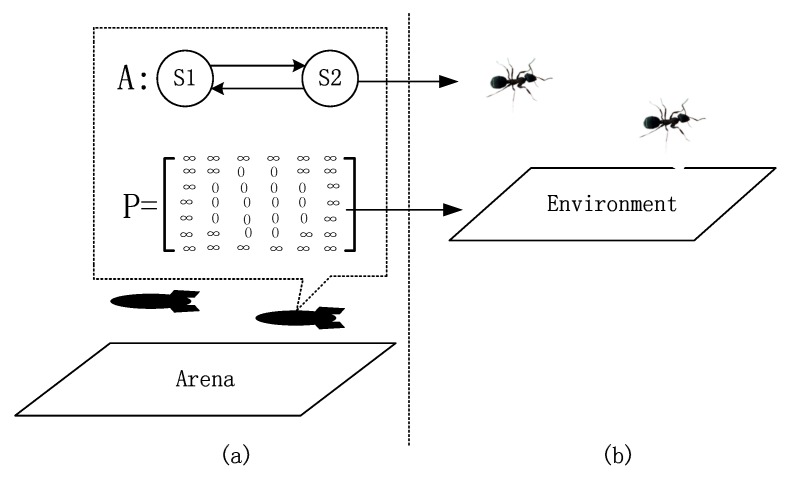
Structure of the controller. (**a**) is a robot swarm monitoring the environment and searching targets in the AOI, mimicking the foraging behavior of ants in (**b**). For each robot, the controller consists of two layers, with *A* being the behavior law and *P* being the pheromone map. The pheromone map is similar to the environment for ants to deploy and sense pheromones. Just like ants can make a decision based on pheromone information, the behavior controller *A* will generate a motion decision based on the pheromone map *P*. It can be seen that all ants in (**b**) share the same environment, but in (**a**) the pheromone maps for different robots differ due to the lack of real-time communication.

To achieve cooperative monitoring and search without direct communication, we design a virtual pheromone-based controller. The controller consists of two layers. The bottom layer is a virtual pheromone map, while the top layer is a behavior controller. The structure is shown in Figure 2. The pheromone map is used to mimic the environment into which an individual can secrete pheromone, and from which an individual can sense pheromones. Thus, this map contains the information of the whole swarm. However, as robots in the swarm do not share real-time communication, the information is readily outdated. Each robot maintains a pheromone map that updates at each step. When visiting a communication node, a robot uploads its pheromone map to the communication network, and then the pheromone map is merged with the one that is maintained by the communication network. Finally, the robot downloads a new pheromone map from the communication network. The behavior controller takes the pheromone map as input, making a decision to guide the robot to voyage in the area, monitoring the environment, searching for targets and visiting the communication nodes.

For robot *i*, take $P_{i}\left(t\right)$ as the pheromone map it maintained at *t*, $B_{i}\left(t\right)$ as its behavior at *t*, and A(·) as the behavior law. $f_{update}\left(\cdot \right)$ is the rule to update the pheromone map and $f_{merge}\left(\cdot \right)$ is the rule to emerge pheromone of itself. The rule maintained by the communication network is defined as $P_{net}\left(t\right)$. The mathematical model of the method can be described with the following functions:

(1) $$B_{i}\left(t\right)=A\left(P_{i}\left(t\right)\right),$$

(2) $$P_{i}\left(t+1\right)=\left\{\begin{array}{cc} f_{merge}\left(P_{i}\left(t\right),P_{net}\left(t\right)\right), & \mathrm{visiting}\;\mathrm{node},\\ f_{update}\left(B_{i}\left(t\right),P_{i}\left(t\right)\right), & \mathrm{otherwise}. \end{array}\right.$$

A robot can calculate its new behavior with Equation (1) based on the pheromone map, and the pheromone map will be updated using Equation (2). In following sections, we will introduce how to define the pheromone map with a matrix in detail. The designs of A(·), $f_{update}\left(\cdot \right)$ and $f_{merge}\left(\cdot \right)$ are also presented.

From the bionic perspective, the behavior controller can be treated as a social insect such as an ant, and the pheromone map it maintains is its environment. The main difference between our robot swarm and a real insect swarm is that, in our swarm, the environment is not real-time. In an ideal situation, namely all robots keep communicating with the communication network, the pheromone map maintained by the robots will be the same and it can reflect the current global situation. However, as robots can only exchange data with the network when visiting a communication node, in general, the pheromone maps maintained by robots are different and outdated. This metaphor is also represented in Figure 2.

## 4. Pheromone Map

We define a pheromone map to mimic the environment for social insect swarms. Agents in the swarm can write information into the pheromone map and read information out. To make it easy to manipulate the pheromone map with mathematical tools, we represent the pheromone map with a m×n matrix *P* as:

(3) $$P={\left(p_{i,j}\right)}_{m\times n},$$

where $p_{i,j}$ implies the pheromone density in $D_{i,j}$, which is a portion of the AOI. The definition of $D_{i,j}$ is given in Section 4.1.

### 4.1. Mapping the AOI into the Pheromone Matrix

**Figure 3:**
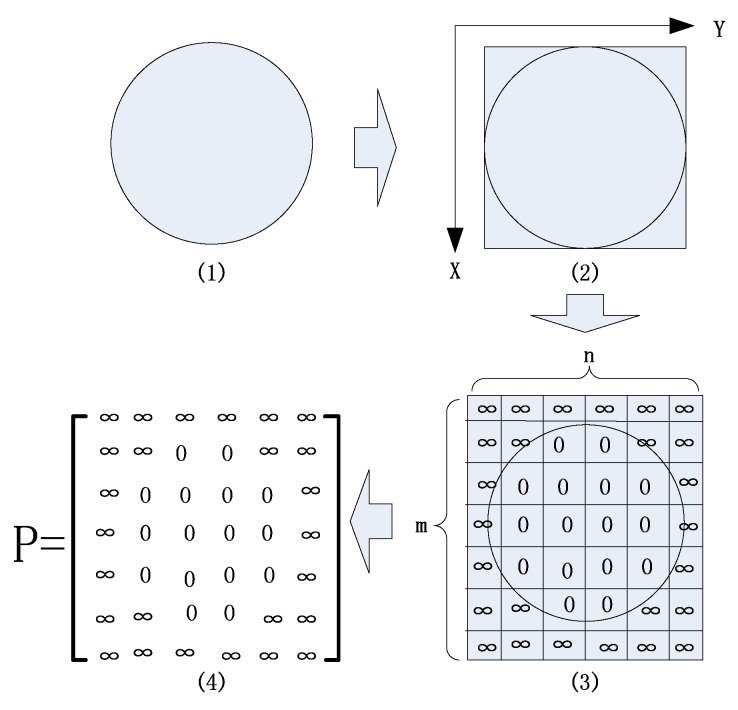
Mapping an AOI into a pheromone matrix. From (1) to (2), we find the MBR of the AOI. The definition of the coordinate system is also shown in (2). Then, the MBR is expanded slightly and scattered into m×n squares in (3). The cells that overlap with the AOI are set to 0, while other cells are set to ∞. This manipulation ensures that, in the pheromone matrix, all inaccessible cells are filled with a repellent pheromone of extremely high density. Finally, we get the pheromone matrix *P* from these cells.

Let the AOI of random shape be a collection of points. We use a point set $D_{AOI}$ to represent the AOI, where each tuple $\left(x,y\right)\in D_{AOI}$ is a point in the AOI. The definition of the coordinate system is shown in Figure 3(2).

We use mapping $f_{ap}:D_{AOI}\to P$ to get the pheromone matrix *P*. Mapping $f_{ap}$ can is represented with Algorithm 1.

The idea of Algorithm 1 is that:

*STEPS 1 and 2*: Build minimum bounding rectangle (MBR).

By finding $X_{\mathrm{MAX}}$, $X_{\mathrm{MIN}}$, $Y_{\mathrm{MAX}}$, and $Y_{\mathrm{MIN}}$ from $D_{AOI}$, we can build a collection

(4) $$D_{MBR}=\left\{\left(x,y\right)|x\in \left[X_{\mathrm{MIN}},X_{\mathrm{MAX}}\right],y\in \left[Y_{\mathrm{MIN}},Y_{\mathrm{MAX}}\right]\right\}.$$

It is an MBR of $D_{AOI}$. In this step, from an AOI with any kind of shape, we can always obtain a rectangle. It is always easy to map a rectangle into a matrix.

*STEPS 3 and 4*: Expand the MBR.

Usually, researchers directly scatter a rectangle to get a matrix. When the rectangle is scattered, we can get a set of rectangles. Then, we can build a matrix with each element representing the pheromone information in each rectangle. This idea is straightforward and is adopted by various research works [48,50,53]. The main drawback of this idea is that special behavior laws are necessary to prevent robots from going out of the AOI.

**Algorithm 1** Mapping $D_{AOI}$ into *P*
**Input:** The AOI: $D_{AOI}$ **Output:** The pheromone matrix: $P={\left(p_{i,j}\right)}_{m\times n}$1:$X_{\mathrm{MAX}}=max\left(x\right)$, $X_{\mathrm{MIN}}=min\left(x\right)$, $Y_{\mathrm{MAX}}=max\left(y\right)$, $Y_{\mathrm{MIN}}=min\left(y\right)$, where $\left(x,y\right)\in D_{AOI}$2:$D_{MBR}=\left\{\left(x,y\right)|x\in \left[X_{\mathrm{MIN}},X_{\mathrm{MAX}}\right],y\in \left[Y_{\mathrm{MIN}},Y_{\mathrm{MAX}}\right]\right\}$3:$X_{\mathrm{MAX}}^{\prime }=X_{\mathrm{MAX}}+\Delta x$, $X_{\mathrm{MIN}}^{\prime }=X_{\mathrm{MIN}}-\Delta x$, $Y_{\mathrm{MAX}}^{\prime }=Y_{\mathrm{MAX}}+\Delta y$, $Y_{\mathrm{MIN}}^{\prime }=Y_{\mathrm{MIN}}-\Delta y$, where $\Delta x>\left(X_{\mathrm{MAX}}-X_{\mathrm{MIN}}\right)/\left(2m-2\right)$, $\Delta y>\left(Y_{\mathrm{MAX}}-Y_{\mathrm{MIN}}\right)/\left(2n-2\right)$4:$D=\{\left(x,y\right)|x\in \left[X_{\mathrm{MIN}}^{\prime },X_{\mathrm{MAX}}^{\prime }\right],y\in \left[Y_{\mathrm{MIN}}^{\prime },Y_{\mathrm{MAX}}^{\prime }\right]\}$5:Scatter *D* into m×n rectangles. Label them with $D_{i,j}$ where i=1,2,…,m, and j=1,2,…,n. Calculate the center of each rectangle, with the center of $D_{i,j}$ being $\left(x_{c}\left(i\right),y_{c}\left(j\right)\right)$:
$$x_{c}\left(i\right)=X_{\mathrm{MIN}}^{\prime }+\frac{2i-1}{2m}\times \left(X_{\mathrm{MAX}}^{\prime }-X_{\mathrm{MIN}}^{\prime }\right)$$,
$$y_{c}\left(j\right)=Y_{\mathrm{MIN}}^{\prime }+\frac{2j-1}{2n}\times \left(Y_{\mathrm{MAX}}^{\prime }-Y_{\mathrm{MIN}}^{\prime }\right)$$.6:Create matrix $P={\left(p_{i,j}\right)}_{m\times n}$
$$p_{i,j}=\left\{\begin{array}{cc} \infty , & \left(x_{c}\left(i\right),y_{c}\left(j\right)\right)\in D_{AOI},\\ 0, & \left(x_{c}\left(i\right),y_{c}\left(j\right)\right)\notin D_{AOI}. \end{array}\right.$$


With our idea, if the AOI is surrounded by repellent pheromone with extremely high density, a robot will not set a point outside of the AOI as its next waypoint. Taking the waypoint as input, a low-level Proportional-Integral-Differential (PID) controller controlling the thruster and rudder will drive a robot back to the AOI in case it is pushed out by water flow. To achieve this, we expand the $D_{MBR}$ into a larger rectangle

(5) $$D=\{\left(x,y\right)|x\in \left[X_{\mathrm{MIN}}^{\prime },X_{\mathrm{MAX}}^{\prime }\right],y\in \left[Y_{\mathrm{MIN}}^{\prime },Y_{\mathrm{MAX}}^{\prime }\right]\}.$$

In *D*, the edges are adjusted into: $X_{\mathrm{MAX}}^{\prime }=X_{\mathrm{MAX}}+\Delta x$, $X_{\mathrm{MIN}}^{\prime }=X_{\mathrm{MIN}}-\Delta x$, $Y_{\mathrm{MAX}}^{\prime }=Y_{\mathrm{MAX}}+\Delta y$, $Y_{\mathrm{MIN}}^{\prime }=Y_{\mathrm{MIN}}-\Delta y$.

Δx and Δy are parameters that should be chosen properly.

*STEP 5*: Scatter *D*.

As we want to obtain an m×n matrix, we scatter the rectangle *D* into m×n cells. We label these cells with $D_{i,j}$ so that $p_{i,j}$ in matrix *P* can represent the pheromone information in $D_{i,j}$. We have

(6) $$D_{i,j}=\left\{\left(x,y\right)|x\in R_{x},y\in R_{y}\right\},$$

(7) $$R_{x}=\left[X_{\mathrm{MIN}}^{\prime }+\frac{i-1}{m}\times \left(X_{\mathrm{MAX}}^{\prime }-X_{\mathrm{MIN}}^{\prime }\right),X_{\mathrm{MIN}}^{\prime }+\frac{i}{m}\times \left(X_{\mathrm{MAX}}^{\prime }-X_{\mathrm{MIN}}^{\prime }\right)\right],$$

(8) $$R_{y}=\left[Y_{\mathrm{MIN}}^{\prime }+\frac{j-1}{n}\times \left(Y_{\mathrm{MAX}}^{\prime }-Y_{\mathrm{MIN}}^{\prime }\right),Y_{\mathrm{MIN}}^{\prime }+\frac{j}{n}\times \left(Y_{\mathrm{MAX}}^{\prime }-Y_{\mathrm{MIN}}^{\prime }\right)\right].$$

The center of $D_{i,j}$ is defined as $\left(x_{c}\left(i\right),y_{c}\left(j\right)\right)$, which can be calculated with:

(9) $$x_{c}\left(i\right)=X_{\mathrm{MIN}}^{\prime }+\frac{2i-1}{2m}\times \left(X_{\mathrm{MAX}}^{\prime }-X_{\mathrm{MIN}}^{\prime }\right),$$

(10) $$y_{c}\left(j\right)=Y_{\mathrm{MIN}}^{\prime }+\frac{2j-1}{2n}\times \left(Y_{\mathrm{MAX}}^{\prime }-Y_{\mathrm{MIN}}^{\prime }\right).$$

We further have

(11) $$x_{c}\left(i\right)=X_{\mathrm{MIN}}-\Delta x+\frac{2i-1}{2m}\times \left(X_{\mathrm{MAX}}-X_{\mathrm{MIN}}+2\Delta x\right),$$

(12) $$y_{c}\left(j\right)=Y_{\mathrm{MIN}}-\Delta y+\frac{2j-1}{2n}\times \left(Y_{\mathrm{MAX}}-Y_{\mathrm{MIN}}+2\Delta y\right).$$

*STEP 6*: Create the pheromone matrix *P*

Finally, we set initial values for each $p_{i,j}$ according to the situation whether $D_{i,j}$ is inside $D_{AOI}$. We treat $D_{i,j}$ inside $D_{AOI}$ if its center $\left(x_{c}\left(i\right),y_{c}\left(j\right)\right)$ is inside $D_{AOI}$, and then set $p_{i,j}$ as 0, namely, no pheromone exists in this cell. Otherwise, the $D_{i,j}$ is out of $D_{AOI}$, which means we do not want a robot moves in $D_{i,j}$. In this case, we set $p_{i,j}$ as ∞, indicating that this cell is filled with repellent pheromone with extremely high density.

To avoid a robot moving out of $D_{AOI}$, we want $D_{AOI}$ surrounded by repellent pheromone. This means:

(13) $$\left(x_{c}\left(1\right),y_{c}\left(j\right)\right)\notin D_{AOI},\left(x_{c}\left(m\right),y_{c}\left(j\right)\right)\notin D_{AOI},\forall j=1,2,\ldots ,n,$$

(14) $$\left(x_{c}\left(i\right),y_{c}\left(1\right)\right)\notin D_{AOI},\left(x_{c}\left(i\right),y_{c}\left(n\right)\right)\notin D_{AOI},\forall i=1,2,\ldots ,m.$$

We have:

(15) $$x_{c}\left(1\right)<X_{\mathrm{MIN}},x_{c}\left(m\right)>X_{\mathrm{MAX}},$$

(16) $$y_{c}\left(1\right)<Y_{\mathrm{MIN}},y_{c}\left(n\right)>Y_{\mathrm{MAX}}.$$

Finally, we can obtain:

(17) $$\Delta x>\left(X_{\mathrm{MAX}}-X_{\mathrm{MIN}}\right)/\left(2m-2\right),$$

(18) $$\Delta y>\left(Y_{\mathrm{MAX}}-Y_{\mathrm{MIN}}\right)/\left(2n-2\right),$$

which is the condition to choose Δx and Δy in *STEP 2*. The whole process is also shown in Figure 3.

### 4.2. Rules to Update Pheromone Matrix

For environment monitoring and static target search applications, we define the density of virtual pheromone as:

(19) $$p_{i,j}\left(t+1\right)=p_{i,j}\left(t\right)+g_{deployment}\left(r_{pos}\left(t\right)\right),$$

(20) $$g_{deploy}\left(t\right)=\left\{\begin{array}{cc} k, & r_{pos}\left(t\right)\in D_{i,j},\\ 0, & r_{pos}\left(t\right)\notin D_{i,j}, \end{array}\right.$$

where $r_{pos}\left(t\right)$ is the position of the robot at *t*. $D_{i,j}$ is the cell corresponding to $p_{i,j}$, and *k* is a parameter indicating the density of the pheromone deployed.

When a robot visits a communication node, it will exchange data with the communication network, merging the matrices maintained by the robot and the communication network, as $f_{merge}\left(\cdot \right)$ in Equation (2).

Assume that one robot has visited a communication node at *T* and visits the communication network again at $T+t_{back}$. Then, $t_{back}$ is defined as the re-visit time. We define a trajectory matrix $M_{trace}={\left(m_{i,j}\right)}_{m\times n}$, whose dimension is the same as that of *P*, to record the trajectory of the robot during $\left[T,T+t_{back}\right]$. In $M_{trace}$, $m_{i,j}=1$ if $D_{i,j}$ has been visited by the robot during $\left[T,T+t_{back}\right]$, otherwise it is set as 0. When visiting a communication node, a robot first uploads the change of the pheromone map caused by its behavior during $\left[T,T+t_{back}\right]$, and then downloads the whole pheromone map. The rules are as follows.

Let $P_{r}$ be the pheromone matrix maintained by the robot before exchanging data, $P_{net}$ be the matrix maintained by the communication network before exchanging data, $P_{r}^{\prime }$ be the matrix maintained by the robot after exchanging data, and $P_{net}^{\prime }$ be the pheromone map maintained by the communication network after exchanging data. $M_{trace}$ is updated to $M_{trace}^{\prime }$ after exchanging data. Then, we have Algorithm 2.

**Algorithm 2** Update pheromone matrix when visiting a communication node**Input:**$P_{r}$, $P_{net}$, $M_{trace}$ **Output:**$P_{r}^{\prime }$, $P_{net}^{\prime }$, $M_{trace}^{\prime }$1:$P_{net}^{\prime }\leftarrow P_{net}+P_{r}\;.*M_{trace}$2:$P_{r}^{\prime }\leftarrow P_{net}^{\prime }$3:$M_{trace}^{\prime }\leftarrow 0$

At Step 2, the operator .∗ is element-wise multiplication. It will return a matrix the same size as the two operands, by multiplying operands’ elements with the same subscript. In this way, we can merge the pheromone map from the robot and that maintained by the communication network.

## 5. Environment Monitoring and Target Search

This section introduces the behavior law to monitor the environment and search for a set of static targets with an underwater robot swarm. It can be treated as an abstraction of deploying a swarm of underwater robots to monitor the AOI and search for underwater mine resources or wreckage after a shipwreck. We also explore to improve the performance by adjusting $t_{back}$ dynamically using a swarm evolution strategy.

### 5.1. Behavior Law for Environment Monitoring and Target Search

**Figure 4:**
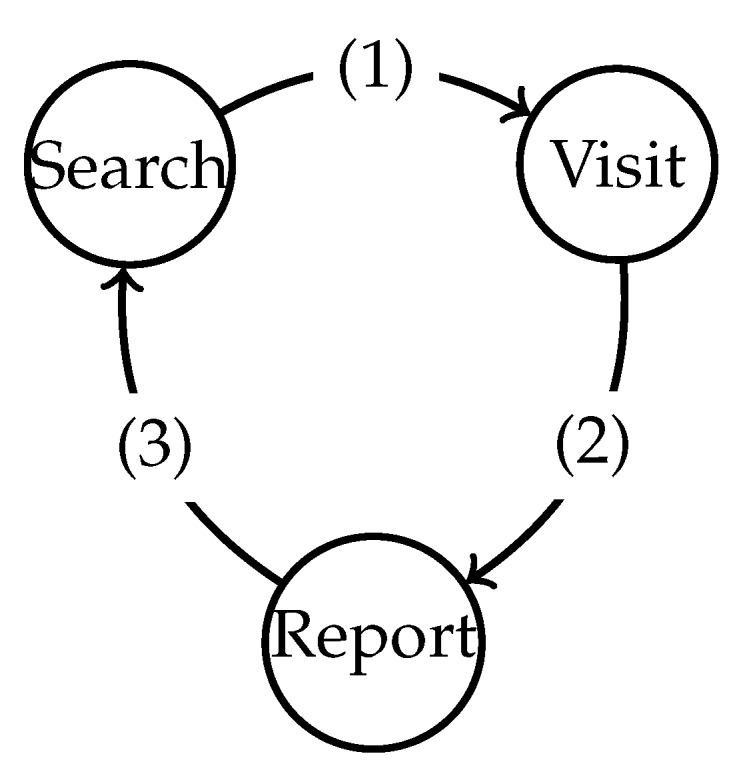
Behavior law to monitor the environment and search for static targets.

The behavior law is represented with a finite state machine, as illustrated in Figure 4. The state transition conditions are given in Table 1. The finite state machine consists of three states: the search state, the visit state, and the report state.

In the search state, robots move in the AOI while monitoring and searching simultaneously. The behavior law is A(·) in Equation (1), which can be treated as a mapping:

(21) $$f_{behavior}:P\to C_{D},$$

(22) $$C_{D}=\left\{D_{i+a,j+b}|a,b\in \left\{-1,0,1\right\}\right\},$$

with the current position of the robot $r_{pos}\left(t\right)\in D_{i,j}$, namely, the robot is currently in $D_{i,j}$. In the next step, it should move to a neighbor cell in $C_{D}$, according to the pheromone matrix *P*.

The intuitive behavior law is to directly mimic behaviors of social insects. In the next step, the robot should directly go to the neighbor cell with the lowest pheromone, i.e., the cell $D_{i*,j*}$, following behavior law:

(23) $$f_{behavior}\left(P\right)=D_{i*,j*},$$

where i∗, j∗ fulfill:

(24) $$p_{i*,j*}=min\left\{p_{i+a,j+b}|a,b\in \left\{-1,0,1\right\}\right\}.$$

When a robot has monitored the environment and searched for a period $t_{back}$, it will change to the visit state.

In the visit state, a robot moves directly to the nearest communication node. When the robot reaches the communication node, it changes to the report state, exchanging data with the communication network following Algorithm 2.

The control commands here and those in the following sections are derived from a discrete model, i.e., the robots are required to track a series of waypoints rather than following continuous velocity commands. This is because the behavior of an underwater robot is disturbed by unpredictable ocean current, and hydrodynamics needs to be taken into account, making it difficult to build an accurate model. The solution to this problem is to adopt a layered controller in underwater robots. The top layer generates commands such as velocity and waypoints, while the bottom layer—usually following PID law—directly controls the steering and thrust. It is usually more effective for underwater robots to track waypoints than following continuous velocity commands because the bottom controller has disturbance rejection capabilities.

Two factors affect the performance of the method. The first factor is the re-visit time $t_{back}$, and the second factor is the behavior law $f_{behavior}$. We explore the effects of the two factors in Section 5.2 and Section 5.3.

### 5.2. The Relationship between $t_{back}$ and Performance

As defined in Section 4.2, $t_{back}$ indicates the re-visit time of a node. We observe that, as $t_{back}$ increases, the performance, namely the coverage rate, for the same swarm first increases and then decreases, as shown by simulation results using different $t_{back}$ in Section 6.

We provide a qualitative explanation for the phenomenon. For a swarm with *N* robots and operation time $T_{total}$, the coverage rate is $P_{c}=c_{visited}\setminus c_{total}$, with $c_{visited}$ being cells that have been visited by at least one robot, and $c_{total}$ the total number of cells in the AOI. As $c_{total}$ is constant, the coverage rate is determined by $c_{visited}$:

(25) $$c_{visited}=\sum_{i=1}^{N}\left(T_{search}^{i}\times \left(1-P_{overlap}^{i}\right)\right),$$

with $T_{search}^{i}$ being the time consumed by robot *i* to search, and $P_{overlap}^{i}$ the possibility of robot *i* visiting a cell that has already been visited. To simplify the analysis, it is assumed that a robot can only visit one cell within a unit time.

Assuming that, during $T_{total}$, robot *i* visits the communication node $N_{b}$ times, then we have $T_{total}=N_{b}\times t_{back}+\sum_{j=1}^{N_{b}}t_{return}^{j}$. Here, $t_{return}^{j}$ is the time for robot *i* to voyage towards the communication node for the *j*th time. As a robot visits the communication node following the shortest path, $t_{return}^{j}<<t_{back}$. To simplify the analysis, we assume that the travel time to the communication node is a constant value $t_{return}$, then we have:

(26) $$T_{total}\approx N_{b}\times \left(t_{back}+t_{return}\right).$$

We further have:

(27) $$N_{b}\times t_{back}\approx T_{total}-N_{b}\times t_{return}.$$

For robot *i*,

(28) $$T_{search}^{i}=N_{b}\times t_{back}.$$

Then, for all robots,

(29) $$T_{search}\approx T_{search}^{i}=T_{total}-N_{b}\times t_{return},$$

(30) $$c_{visited}\approx T_{search}\times \sum_{i=1}^{N}\left(1-P_{overlap}^{i}\right).$$

$c_{visited}$ is determined by $T_{search}$ and $P_{overlap}^{i}$. With the increase of $t_{back}$, $N_{b}$ decreases so that $T_{search}$ increases, i.e., the increase of $t_{back}$ is favorable to the increase of $c_{visited}$. Meanwhile, without real-time data of each other, the chance for a robot to visit a cell that has already been visited by other robots also increases with the increase of $t_{back}$. i.e., $P_{overlap}^{i}$ increases, which is a negative factor for $c_{visited}$. When $t_{back}$ is small, $P_{overlap}^{i}$ is so small that $T_{search}$ plays the main role, and, with the increase of $t_{back}$, the weight of $P_{overlap}^{i}$ increases, and finally neutralizes the advantage brought by $T_{search}$. Thus, the coverage rate first increases and then decreases with the increase of $t_{back}$, as shown in the experimental section.

As the robot swarm is a complex system affected by multiple contradictory factors, we are unable to analyze the effects of $t_{back}$ quantitatively, i.e., unable to theoretically obtain the $t_{back}$ with the best performance. Therefore, a swarm intelligent method is proposed to adjust the parameter $t_{back}$ online and automatically. For robot *r*, we define $p_{back}$ to measure the performance of $t_{back}$:

(31) $$p_{back}=1-|c_{overlap}^{r}\left|/\right|c_{T,T+t_{back}}^{r}|,$$

(32) $$c_{overlap}^{r}=c_{T,T+t_{back}}^{r}\cap c_{T+t_{back}}^{all},$$

where $c_{T,T+t_{back}}^{r}$ represents the cells that have been visited by robot *r* during time range $\left[T,T+t_{back}\right]$, and $c_{T+t_{back}}^{all}$ indicates the cells that have been visited by other robots during this period.

Apparently, $p_{back}$ implies the rate of new cells that have been visited only by robot *r* to the cells visited by robot *r* during the last period. Thus, we have $p_{back}\in \left[0,1\right]$ and, if $p_{back}$ is larger, the performance is better.

Based on $p_{back}$, the robots in the swarm can adjust $t_{back}$ automatically according to the performance during the last $t_{back}$ interval, and the method is as follows:

- Set initial values. For each robot, we assign a small initial value to $t_{back}\left(0\right)$. We also set $p_{back}\left(0\right)=0$, and k(0)=1.
- When a robot visits the communication network the $i^{th}$ time, calculate $p_{back}\left(i\right)$ and update $t_{back}\left(i\right)$
  (33) $$t_{back}\left(i\right)=t_{back}\left(i-1\right)+k\left(i\right)\Delta t,$$
  where
  $$k\left(i\right)=\left\{\begin{array}{cc} k(i-1) & p_{back}\left(i\right)\geq p_{back}\left(i-1\right),\\ -k(i-1) & p_{back}\left(i\right)<p_{back}\left(i-1\right) \end{array}\right.$$
  and Δt is a parameter.

When robots in the swarm adjust their own $t_{back}$ automatically with the method above, we can get an acceptable performance. Even though it is not an optimal solution, we can avoid the risk of choosing an unfavorable $t_{back}$.

### 5.3. Improve Performance by Using Global Information

The behavior law $f_{behavior}$ in Section 5.1 just uses the information from the nine neighbor cells. This strategy wastes the information from far-away cells. A better strategy should consider both nearby and far-away pheromones. In this section, we propose an improved behavior law as follows:

(34) $$f_{behavior}^{\prime }:P\to C_{D},$$

(35) $$C_{D}=\left\{D_{i+a,j+b}|a,b\in \left\{-1,0,1\right\}\right\}.$$

Assume that the current position of the robot is $r_{pos}\left(t\right)\in D_{i,j}$. In order to obtain $f_{behavior}^{\prime }$, we define two 3×3 matrices $P_{local}$ and $P_{global}$. $P_{local}$ is a sub-matrix of *P* as follows:

(36) $$P_{local}=\left[\begin{array}{ccc} p_{i-1,j-1} & p_{i-1,j} & p_{i-1,j+1}\\ p_{i,j-1} & p_{i,j} & p_{i,j+1}\\ p_{i+1,j-1} & p_{i+1,j} & p_{i+1,j+1} \end{array}\right].$$

Apparently, $P_{local}$ implies the pheromone information in cells in $C_{D}$. As cells in the border region are assigned a pheromone whose density is ∞, a robot does not set a point at the border as its next waypoint. Only when it reaches one waypoint will a robot plan the next one. Hence, during the whole searching process, waypoints are within the AOI. As a result, we can always get $P_{local}$.

$P_{global}$ is a matrix compressed from *P*, which means that $P_{global}$ should contain the pheromone information of the whole pheromone matrix. We get $P_{global}$ with Algorithm 3.

**Figure 5:**
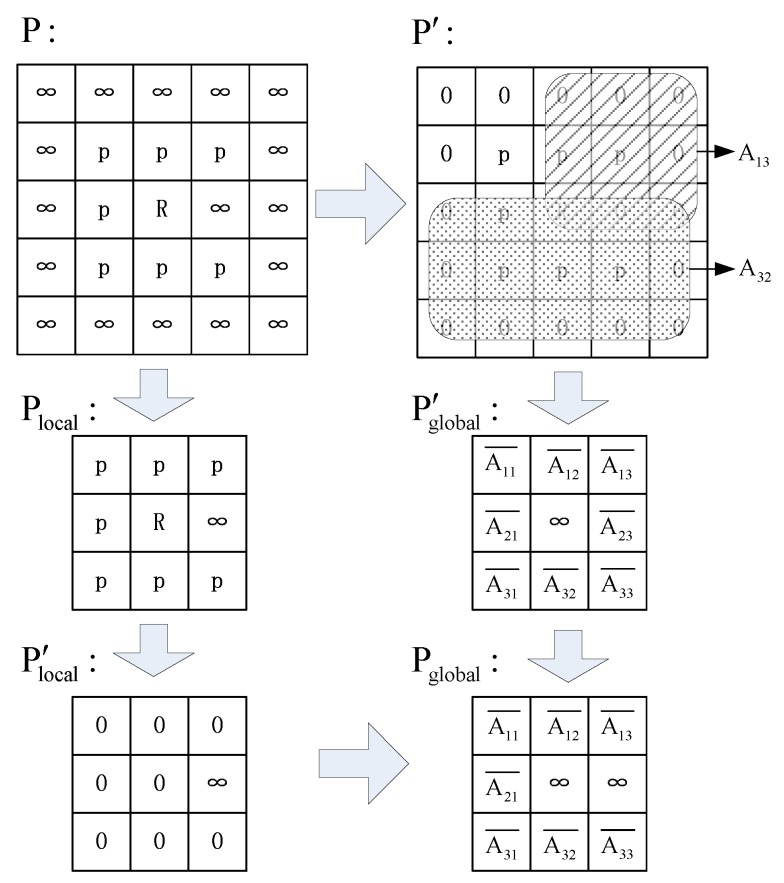
Steps of getting $P_{global}$ from *P*. *R* indicates the current position of the robot, and *p* is pheromone value that is not equal to ∞. ∞ indicates the inaccessible cells. From *P*, we can get $P^{\prime }$ and $P_{local}$. $P^{\prime }$ is compressed into $P_{global}^{\prime }$. $P_{local}$ transits to $P_{local}^{\prime }$ by replacing all elements not equal to ∞ with 0. Finally, we get $P_{global}$ by summing up $P_{local}^{\prime }$ and $P_{global}^{\prime }$. $P_{global}$ implies the average pheromone density at each direction and indicates the adjacent inaccessible cells with ∞.

In *STEP 1*, we replace the ∞ elements in *P* with 0 to get $P^{\prime }$ so that we can sum elements up and calculate the average value. Then, in *STEP 2*, we compress $P^{\prime }$ into a 3×3 matrix. This is achieved by dividing $P^{\prime }$ into nine sub-matrices and calculate the average values of these sub-matrices, as shown in Figure 5. After this step, we can obtain a matrix called $P_{global}^{\prime }$. $P_{global}^{\prime }$ already contains the information of the whole pheromone matrix. However, it does not indicate if the neighbor cells are accessible. Thus, in *STEP 3*, we get a matrix $P_{local}^{\prime }$ that indicates all the inaccessible neighbor cells. In *STEP 4*, we combine $P_{local}^{\prime }$ and $P_{global}^{\prime }$ to get $P_{global}$ that contains the global information, while indicating neighbor inaccessible cells with ∞. The process is shown in Figure 5.

**Algorithm 3** Compress *P* into $P_{global}$
**Input:***P*, $P_{local}$, $r_{pos}\in D_{i,j}$ **Output:**$P_{global}$1:Define $P^{\prime }={\left(p_{i,j}^{\prime }\right)}_{m\times n}$:
$$p_{i,j=}^{\prime }\left\{\begin{array}{cc} 0 & p_{i,j}=\infty \\ p_{i,j} & p_{i,j}\neq \infty \end{array}\right.$$2:Compress matrix $P^{\prime }$ into 3×3 matrix $P_{global}^{\prime }=$
$$\left[\begin{array}{ccc} \frac{\sum_{a=1,b=1}^{a=i,b=j}p_{a,b}^{\prime }}{i\times j} & \frac{\sum_{a=1,b=1}^{a=i,b=n}p_{a,b}^{\prime }}{i\times n} & \frac{\sum_{a=1,b=j}^{a=i,b=n}p_{a,b}^{\prime }}{i\times (n-j+1)}\\ \frac{\sum_{a=1,b=1}^{a=m,b=j}p_{a,b}^{\prime }}{m\times j} & \infty & \frac{\sum_{a=1,b=j}^{a=m,b=n}p_{a,b}^{\prime }}{m\times (n-j+1)}\\ \frac{\sum_{a=i,b=1}^{a=m,b=j}p_{a,b}^{\prime }}{(m-i+1)\times j} & \frac{\sum_{a=i,b=1}^{a=m,b=n}p_{a,b}^{\prime }}{(m-i+1)\times n} & \frac{\sum_{a=i,b=j}^{a=m,b=n}p_{i,j}^{\prime }}{\left(m-i+1)\times (n-j+1\right)} \end{array}\right]$$3:Mapping $P_{local}$ to $P_{local}^{\prime }$ by replacing all elements not equal to ∞ into 04:$P_{global}=P_{local}^{\prime }+P_{global}^{\prime }$

Now, we have two 3×3 matrices, i.e., the local matrix $P_{local}$ and the global matrix $P_{global}$. Then, we can decide the motion of the robot according to the two matrices. The idea is that, if a neighbor cell has not been visited before, the robot will explore it. If all neighbor cells have already been visited, the robot will move in the direction with the lowest pheromone density. We define the next cell to visit as $D_{i*,j*}$, which can be obtained with Algorithm 4.

**Algorithm 4** Get the next cell to visit according to pheromone matrix**Input:**$P_{local}$, $P_{global}$ **Output:**$D_{i*,j*}$ 1:Create collection $C_{global}$ that contains all elements of $P_{global}$ 2:Create collection $C_{local}$ that contains all elements of $P_{local}$ 3:Create empty collection *C* 4:**for**$p_{a,b}$ in $C_{local}$ **do** 5:        **if** $p_{a,b}=0$ **then**
 6:                put (a,b) into *C* 7:        **end if**
 8:**end for** 9:**if**|C|≠0**then**10:        (i∗,j∗)←random(C)
11:**end if**12:**if**|C|=0**then**13:        **for** $p_{a,b}$ in $C_{global}$ **do**14:                **if** $p_{a,b}=min\left(C_{global}\right)$ **then**
15:                        put (a,b) into *C*16:                **end if**
17:        **end for**
18:        (i∗,j∗)←random(C)
19:**end if**

The behavior law means:

- Check local matrix $P_{local}$ and go to a random cell whose pheromone density is 0.
- If no cell in $P_{local}$ equals 0, move to the cell with the lowest element in $P_{global}$.

## 6. Simulation and Real-World Experiment

Simulations are carried out in Matlab (version 2014a) to test the strategy proposed in this paper. We use an underwater robot swarm to monitor the environment and search for static targets. The proposed methods are evaluated, and factors affecting the performance of the methods are analyzed. Finally, we give some recommendations for the application of the methods according to the simulation results.

### 6.1. Simulation

In the simulation of monitoring the environment and searching for static targets, we set the AOI as a rectangle composed of 200×200 cells. At each step, a robot can move to one of the adjacent cells, and the total simulation time is set to 2000 steps. We assume that a robot is able to move from one cell to another in one step. A communication network of sixteen communication nodes is deployed into the AOI. These nodes are deployed evenly into the AOI, forming a uniform grid. All robots are deployed from the same position. This is because, in practical application, all robots in the swarm are deployed by the same mothership or from the same base station. In Section 5, we develop two behavior laws. The behavior law $f_{local}$ in Section 5.1 uses only local pheromone information, while $f_{global}$ in Section 5.3 uses global pheromone information. Both methods are simulated. We also test a random search scheme with all robots moving randomly. In Section 5.2, we assume that the re-visit time $t_{back}$ can affect the performance of the method. Thus, we implement simulations with different $t_{back}$. To eliminate randomness, for the same setting, we repeat the simulation 10 times. Each setting is defined as

$$setting=(number,t_{back},behavior\_law),$$

number∈{10,20,30,40,50},

$$t_{back}\in \left\{50,150,250,350,450,550,650,750,\infty \right\},$$

$$behavior\_law\in \{f_{local},f_{global},f_{random}\}.$$

For a random search, no $t_{back}$ is used.

As all experiments are carried out in the area of the same size, the number of robots can also reflect the density of robots. As the swarm is used to monitor the environment and search static targets, we assume that, only when the target that is within the cell has been visited by a robot, it can be found. Thus, we use the coverage rate at the final time to measure the performance of the method.

**Figure 6:**
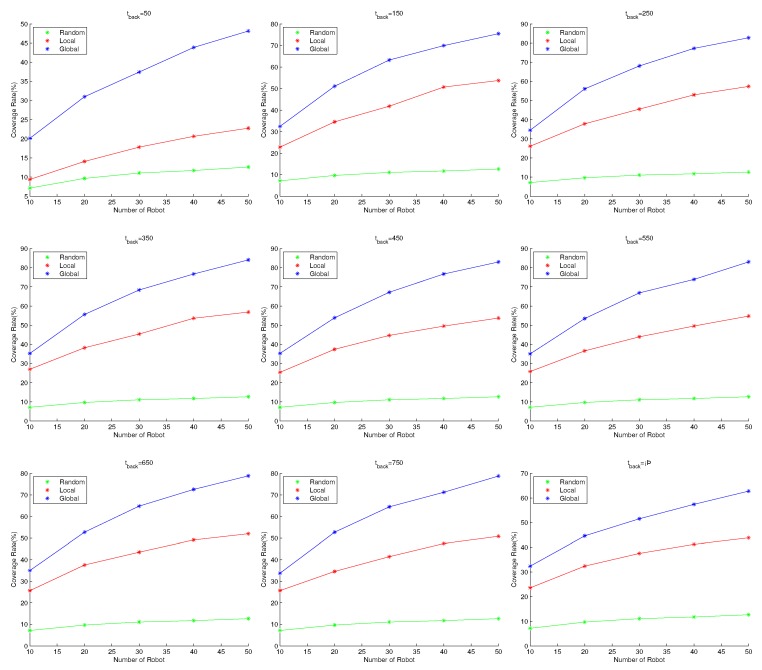
Comparison of performance for $f_{global}$, $f_{local}$ and $f_{random}$. In each subfigure, $t_{back}$ is set with the same value, and swarms with 10, 20, 30, 40, 50 robots are tested with all three behavior laws. The performance of the control law is evaluated with coverage rate. From each subfigure, we notice that, for a fixed number of robots, no matter how $t_{back}$ is set, the performance of $f_{global}$ is always superior to that of $f_{local}$. Both the $f_{global}$ and $f_{local}$ schemes perform better than $f_{random}$. In addition, with the increase of swarm size, the performance also increases no matter which scheme is adopted. However, the increase of performance is obvious for $f_{local}$ and $f_{global}$, while that for $f_{random}$ is rather insignificant. This trend appears in each subfigure, which implies that $f_{global}$ is superior to $f_{local}$ and $f_{random}$ no matter how $t_{back}$ is set.

**Figure 7:**
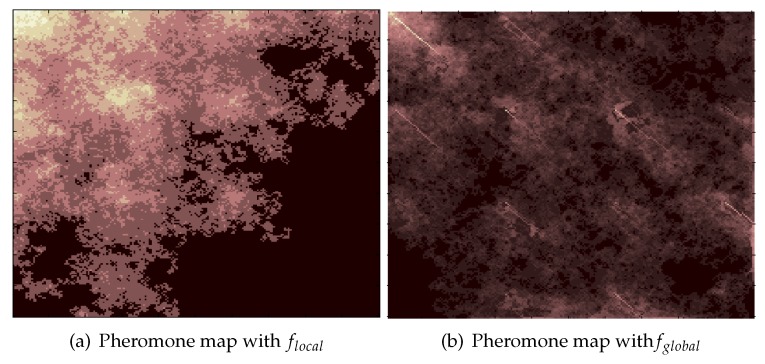
Typical pheromone maps with behavior law $f_{local}$ and $f_{global}$. The pheromone maps are shown with a grayscale map, indicating the density of repellent pheromone. The density of the pheromone is higher in the lighter region and lower in the darker region. The pheromone density is 0 in the black region. We want most areas to be visited by robots, but not repeated many times; (**a**) is not perfect because it has a large black part, representing regions that have never been visited. It also has a large part that is extremely white, indicating that these regions have been visited repeatedly, which is a duplication of label; (**b**) is better because almost the whole AOI is covered with a layer of light white, indicating that most parts have been visited, and not repeated over and over again.

From the simulation results in Figure 6, we compare the performance of the three methods with the same number of robots and the same $t_{back}$. It can be found that the method using the global pheromone information is better than the method using only local pheromone information. The performances of both pheromone-based methods are superior to that of the random search method. We note that, except for $t_{back}=50$, the coverage rate with 20 robots and the behavior law $f_{global}$ is similar to that of a swarm with 50 robots using $f_{local}$. In addition, for $t_{back}=50$, when the size of swarm using $f_{global}$ is 10, the coverage rate is similar to a $f_{local}$ swarm with 50 robots. This means that $f_{global}$ is very superior to $f_{local}$. This is because, by using the $P_{global}$, the strategy $f_{global}$ can guide the robots to the region with a lower repellent pheromone density in which fewer cells have been visited by robots. As a result, with $f_{global}$, the pheromone density is more even and robots can spread out in a short time. Figure 7 provides the pheromone map of a swarm using $f_{local}$ and that of a swarm using $f_{global}$ for comparison. The pheromone maps are represented with a grayscale map, indicating the density of the pheromone. In both cases, the size of the swarm is 50 and the $t_{back}$ is set to 250. It can be seen that, when using $f_{local}$, the grayscale map is more imbalanced. The light region implies that these cells have been visited multiple times, so repellent pheromones have been deployed again and again, while the large dark regions have never been visited. Meanwhile, the map using $f_{global}$ is very balanced, and the dark region is much smaller. This implies that fewer cells have been visited multiple times by the swarm, enabling robots to explore new regions.

**Figure 8:**
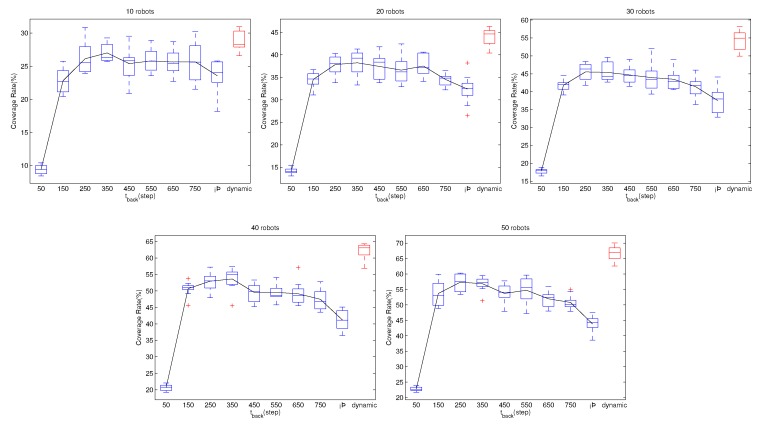
The effects of $t_{back}$ on behavior law $f_{local}$. Each subfigure illustrates the variation of the swarm’s performance with the increase of $t_{back}$ when using a fixed number of robots. The robot numbers are 10, 20, 30, 40, and 50, respectively. The $t_{back}$ increases from 50 to 150, 250, 350, 450, 550, 650, 750, and ∞. With ∞, it means that the robots never visit the communication network. The red box shows the coverage rate with the method in Section 5.2 that adjusts $t_{back}$ automatically. The black line links the mean values of the coverage rate, showing how the performance changes with the increase of $t_{back}$. From the black line, we notice that, with the increase of $t_{back}$, the performance first increases and then decreases. There exists a best $t_{back}$ which provides the fastest coverage rate. However, as analyzed in Section 5.2, we are unable to obtain this value directly. Meanwhile, the red box shows that, with the dynamic adjust $t_{back}$ strategy, the performance is superior to that of any fixed $t_{back}$. The phenomenon appears in each subfigure, i.e., simulation with different swarm size, indicating that, when using $f_{local}$, the dynamic adjust $t_{back}$ scheme performs better than any fixed $t_{back}$.

**Figure 9:**
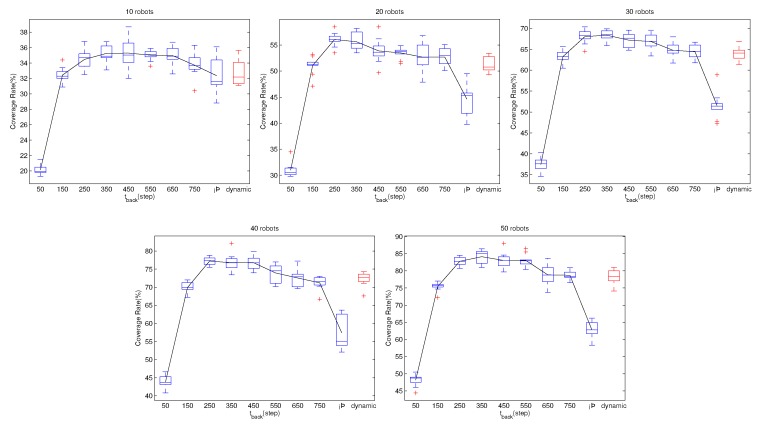
The effects of $t_{back}$ on behavior law $f_{global}$. Each subfigure illustrates the variation of the swarm’s performance with the increase of $t_{back}$ when using a fixed number of robots. The robot numbers are 10, 20, 30, 40, and 50, respectively. The $t_{back}$ increase from 50 to 150, 250, 350, 450, 550, 650, 750, and ∞. With ∞, it means that the robots never visit the communication network. The red box shows the coverage rate with the method in Section 5.2 that adjusts $t_{back}$ automatically. The black line links the mean values of the coverage rate, showing how the performance changes with the increase of $t_{back}$. Similar to Figure 8, in each subfigure, the black line first increases and then decreases, indicating that, with the increment of $t_{back}$, the coverage rate will first increase and then decrease. From the five sub-figures, the red box is a bit lower than the top of the black line. This indicates that, with $f_{global}$, the dynamic adjust $t_{back}$ scheme will provide the performance that is not the best, but still acceptable. Considering that we are unable to get the best $t_{back}$ in advance, using the dynamic adjust $t_{back}$ scheme has application value because it can avoid choosing a poor $t_{back}$.

In Section 5.2, we predict that $t_{back}$ can affect the performance of the swarm, and with the increase of $t_{back}$, the coverage rate will first increase and then decrease. Figure 8 and Figure 9 show the effects of $t_{back}$ to the performance of the swarm. We can clearly see the trend that the performances first increase and then decrease. Therefore, it is important to choose a proper $t_{back}$.

To solve this problem, we propose a method in Section 5.2 that can dynamically adjust the $t_{back}$ for each robot. Again, we use a different number of robots and the two kinds of behavior controllers to perform simulations. In these cases, the $t_{back}$ is not a constant value, and keeps changing following the scheme in Section 5.2. The simulation results are shown in Figure 8 and Figure 9. In each subfigure, the red box shows the performance while automatically adjusting the $t_{back}$. From Figure 8, it is obvious that, when using $f_{local}$, the performance with dynamic $t_{back}$ is better than that of any constant $t_{back}$. From Figure 9, when using $f_{global}$, the performance with dynamic $t_{back}$ is mediocrity. Even though the dynamic $t_{back}$ scheme is not optimal, the performance is acceptable. More importantly, evolving the $t_{back}$ by the swarm, we can avoid the risk of choosing a bad $t_{back}$.

### 6.2. Real-World Experiment

**Figure 10:**
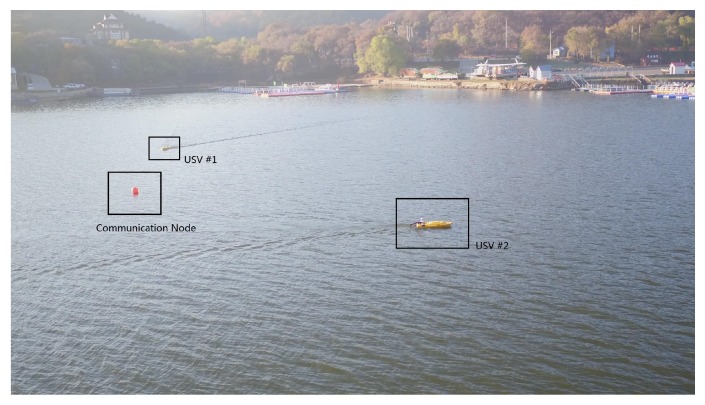
The lake experiment carried out with two USVs and one communication node. USV #1 is approaching the communication node to update its pheromone map, while #2 that has just visited the node is exploring the lake.

**Figure 11:**
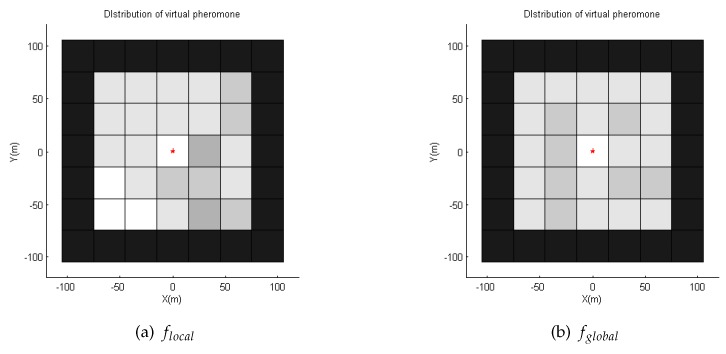
Pheromone distribution with $f_{local}$ and $f_{global}$. In our test, the AOI is scattered into 5×5 grids with Algorithm 1. The color of each square indicates the density of pheromone: the darker a square, the greater the pheromone density is. The edge of the AOI is black and the corresponding pheromone density is ∞, preventing the robots from going out of the AOI. The red star indicates a communication node. With $f_{global}$, pheromone has been deployed into the whole area, but it is not the case when using $f_{local}$. In addition, the density with global is more balanced.

The experiment is carried out with a USV swarm in Xiuhu Lake, Shenyang, China. As experiments with a swarm of underwater robots are costly, it is a common practice to mimic the behavior of underwater robots using USVs as an alternative in real-world experiments, such as in [72,73]. As shown in Figure 10, a float ball acting as the communication node is located at the center of the lake (123.653555 E, 41.934899 N). In the lake, we set a square of 150 × 150 m${}^{2}$ as the AOI and deploy two USVs. The AOI is first mapped into a matrix representing the pheromone with Algorithm 1. This is achieved by scattering the AOI into a set of grids properly. The USVs adopt GPS to obtain their locations and the positioning error is around 5 m. The cruise speed of both USVs are 1.5 m/s, with the turn radius being 10 m. With these parameters, we scatter the AOI into 25 cells, with the edge of each cell being 30 m. This value is big enough for USVs to overcome negative effects caused by positioning errors, and is larger than the turn radius so that one USV can move smoothly from one cell to another without circling around the destination. To prevent the USVs from going out of the AOI, the edge of the AOI is expanded slightly, generating the border region filled with the pheromone of ∞ density. In this case, we set a border region whose width is 30 m, as shown in Figure 11. The two USVs are unable to communicate with each other but can exchange data with a communication node when the distance between them is less than five meters.

In the experiment, two behavior laws are tested, which are based on local pheromone information in Section 5.1 and global pheromone information in Section 5.1, respectively. Considering the choice of $t_{back}$ and the simulation results in Section 6.1, the scheme in Section 5.2 can adjust $t_{back}$ dynamically, providing an acceptable performance in both cases. Thus, in the test, we do not set a fixed $t_{back}$ but rather adjust it dynamically. The performance is evaluated with the distribution of pheromones in the area, and we are interested in two metrics. The first metric is the coverage rate, implying if the whole area has been explored by the swarm. The second metric is the mean square error of the density of pheromone. A small mean square error indicates that each square is visited frequently enough, enabling the robots to find emerging targets in time.

Both methods are tested for 20 min, with Figure 11 showing the distribution of pheromones for both methods. With the local pheromone-based method, the coverage rate is 88% with the mean square error of 0.3594. However, with the global pheromone-based method, the coverage rate reaches 100% with a smaller mean square error of 0.2899. We conclude that the scheme adopting global pheromone information performs better. This result further supports the discussion in Section 5 and verifies the simulation results.

## 7. Conclusions

An underwater robot swarm can be applied for maritime monitoring applications. Compared with a single robot, a swarm covers a larger area and it can accomplish tasks more rapidly by working collectively. In this paper, we propose a monitoring strategy for an underwater robot swarm. The use of robots makes it possible to monitor a dynamically selected area, rather than monitor a fixed area using stationary monitoring sensors. Our strategy deals two aspects, i.e., communication and swarm monitoring behavior. We build a communication network which contains a set of underwater communication nodes. Robots periodically visit communication nodes to exchange information with each other in an indirect way. To form cooperative swarm monitoring behavior, we apply a pheromone-inspired controller to each robot. The controller uses virtual pheromone to store the information of an AOI. Behavior laws are designed to guide robots to monitor the environment with the help of the virtual pheromone. In the monitoring process, static target search—such as wreckage or mine resources—can be performed simultaneously. Once the targets are found, they can be reported by updating information to communication nodes.

Experimental results indicate that, among the three schemes we tested (i.e., $f_{global}$, $f_{local}$ and random search), the $f_{global}$ scheme performs best, and both $f_{global}$ and $f_{local}$ schemes work better than the random search scheme. This can be explained from the degree of "cooperation" among robots. With the random search scheme, every robot works independently. Thus, they do not use information from their peers, resulting in duplicate work. With $f_{local}$, a robot uses the nearby information. It can infer the density of nearby robots and visit unexplored areas. With $f_{global}$, a robot can obtain the information of the whole AOI. The more information it has, the wiser a decision it can make.

There is a trade-off between performance and computation cost. For a robot swarm with a random search scheme, only a few robots are needed and the calculation workload is small. When adopting a $f_{local}$ scheme, an underwater communication network consisting of a set of communication nodes is necessary. To achieve better performance, we apply $f_{global}$ scheme, which may require a high performance computer because a robot needs to handle a matrix representing the whole AOI. With the expansion of the AOI, the calculation workload will also increase. If the AOI is enormous, robots may need a high performance computer, which will increase the cost of the swarm, as well as the energy consumption. However, with $f_{local}$, no matter how large the AOI is, the calculation workload is fixed and small because only a 3×3 matrix is processed (or 3×3×3 matrix when the third dimension is added). As a result, the robot can carry a computer with lower performance, thus reducing the cost of the robot.

In addition, the performance of $f_{global}$ can be further improved. The key point is to extract valuable information from the pheromone matrix. Our current scheme uses the mean pheromone density in each direction to determine the behavior of a robot. However, from the global matrix, other information can also be used. A proper choice of the information may improve the performance of the swarm, and that is what we are currently working on.

In order to achieve a reasonable performance, we introduce an evolution scheme that automatically varies the visiting period of the robots in the monitoring process. Simulation results reveal that, for $f_{local}$, the performance of dynamic $t_{back}$ is superior to that of any fixed $t_{back}$. However, for $f_{global}$, the performance of this scheme is not remarkable. This is because a robot adjusts its $t_{back}$ based on its own historical performance. There is a chance that the strategy can be improved by utilizing the historical performance of other robots.

In the future, we plan to enhance the performance of our monitoring strategy by predicting global information based on past information from the network and demonstrate its effectiveness in real-world applications.

## Figures and Tables

**Table 1:** State transition condition in Figure 4 for static targets.

Marker	Description
(1)	Monitor the environment and search for a period of $t_{back}$
(2)	Reach the nearest communication node
(3)	Finish exchanging data with the communication node
